# Detection of *BRAF* mutations from solid tumors using Tumorplex™ technology

**DOI:** 10.1016/j.mex.2015.06.002

**Published:** 2015-06-12

**Authors:** Jacob Yo, Katie S.L. Hay, Dilanthi Vinayagamoorthy, Danielle Maryanski, Mark Carter, Joseph Wiegel, Thuraiayah Vinayagamoorthy

**Affiliations:** MultiGEN Diagnostics LLC, 854 Paragon Way, Rock Hill, SC 29730, United States

**Keywords:** Multiplex Sanger sequencing platform, Tumorplex™, *BRAF* V600E, Tumors, Lowest level of detection, Formalin fixed paraffin embedded, Weighted sequencing primers, Multiplex Sanger sequencing platform

## Abstract

Allele specific multiplex sequencing (Tumorplex™) is a new molecular platform for the detection of single base mutation in tumor biopsies with high sensitivity for clinical testing. Tumorplex™ is a novel modification of Sanger sequencing technology that generates both mutant and wild type nucleotide sequences simultaneously in the same electropherogram. The molecular weight of the two sequencing primers are different such that the two sequences generated are separated, thus eliminating possible suppression of mutant signal by the more abundant wild type signal. Tumorplex™ platform technology was tested using BRAF mutation V600E. These studies were performed with cloned BRAF mutations and genomic DNA extracted from tumor cells carrying 50% mutant allele. The lower limit of detection for BRAF V600E was found to be 20 genome equivalents (GE) using genomic DNA extracted from mutation specific cell lines. Sensitivity of the assay was tested by challenging the mutant allele with wild type allele at 20 GE, and was able to detect BRAF mutant signal at a GE ration of 20:1 × 10^7^ (mutant to wild-type). This level of sensitivity can detect low abundance of clonal mutations in tumor biopsies and eliminate the need for cell enrichment.

• Tumorplex™ is a single tube assay that permits the recognition of mutant allele without suppression by wildtype signal.
• Tumorplex™ provides a high level of sensitivity.
• Tumorplex™ can be used with small sample size with mixed population of cells carrying heterogeneous gDNA.

## Graphical abstract

**Figure:**
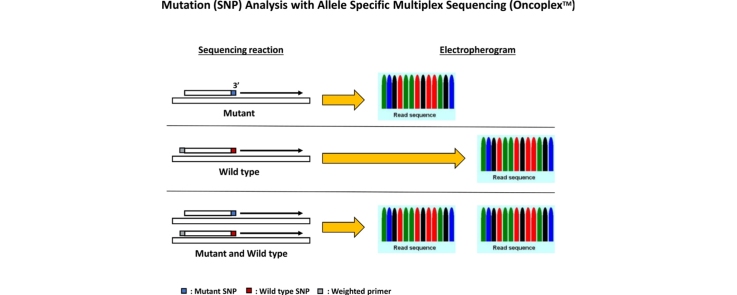

## Method details

### Primer design

A set of PCR primers BRAF-UP: 5′-AACTCTTCATAATGCTTGCTCTGA-3′ and BRAF-LP: 5′-CAGACAACTGTTCAAACTGATGGGACC-3′ were used to amplify a region of human gDNA encompassing the *BRAF* V600 locus. The ampliﬁed product was 180 base pairs (bp). Two sequencing primers were designed to recognize the respective single nucleotide at their 3′ end independently, one for *BRAF* mutant V600E and the other for *BRAF* wild type V600. These two sequencing primers differed in two respects: the nucleotide at their 3′ end and the respective molecular weight. Allele specific nucleotide at their 3′ end determined their respective specificity of the two sequence primers. e.g., *BRAF* V600E mutant harbors a deoxythymidine and V600 wild-type carries a deoxyadenosine. The different molecular weights of the allele-speciﬁc sequencing primer separated the truncated molecules generated from the mutant sequencing primer from the wild type sequencing primer. Allele-speciﬁc sequencing primers used were (mutant) V600E-SP: 5′-AATAGGTGATTTTGGTCTAGCTACAGT-3′ and (wild type) V600-SP: 5′-weighted-AATAGGTGATTTTGGTCTAGCTACAGA-3′.

### Analytical validation

The analytical validation for *BRAF* V600E was performed using mutant or wild-type plasmid clone DNA (GENEWIZ, USA) and genomic DNA (gDNA) (Horizon Diagnostics, UK) extracted from cell lines harboring both the mutation and wild-type (50:50) alleles. The lower limit of detection (LLOD) was determined by serial dilutions of gDNA with 10 mM Tris, 1 mM EDTA (TE).

### Criterion for determining a positive result

A positive result is determined by two criteria: the correct nucleotide sequence and the correct position (data point) on the electropherogram. The wild-type and mutant have the same nucleotide sequence requirement; a “C” must be followed by “TGTAGCTAGA”. The positive wild-type signal also functions as an “internal process control” and should always be detected at its correct position with its correct nucleotide sequence. It should be noted that at low levels of detection, one may not see all of the expected sequence. Therefore we recommend that the minimum number of nucleotides to be six (CTGTAG). With this level of criteria, the probability of the nucleotide sequence CTGTAG being found is 1/4^6^, or 1 in 4096.

### Sample preparation

1. The region encompassing the V600E mutation was amplified using either cloned DNA or gDNA.
2. Each reaction mixture included 25 μl of Master Mix 2X buffer (Multiplex PCR Plus Kit, Qiagen, USA), 1 μl each of forward and reverse primers at 10 μM, 1 or 2 μl of template, and volume brought up to 50 μl with TE. For cross-reactivity assays, clonal templates of either 1 μl 0.6 ng/μl *BRAF* V600E, 1 μl 0.55 ng/μl *BRAF* V600, or 1 μl of both were used. For LLOD testing, 1 μl of respective dilution from stock *BRAF* V600E gDNA at 50% allelic frequency (9.36 ng/μl) was used. For sensitivity determination, 1 μl of clonal *BRAF* V600 respective dilution was used to challenge 1 μl of the LLOD previously found.
3. PCR conditions consisted of an initial denaturation at 95 °C for 5 min; 35 cycles of 95 °C for 30 s, 55 °C for 90 s, and 72 °C for 30 s; and a final extension at 68 °C for 10 min.
4. After PCR, 5 units of Uracil-DNA glycosylase were added to the reaction and incubated for 60 min at 37 °C and deactivated at 96 °C for 3 min.
5. The amplicons were cleaned using AMPure according to manufacturer’s instruction (Beckman Coulter, USA).
6. Purified amplicons (2.5 μl) were sequenced in a 10 μl reaction volume using 1 μl allele-specific (*BRAF* V600E or V600) sequencing primers (MultiGEN Diagnostics, LLC, USA) at 3.3 μМ each, 1 μl of ABI PRISM BigDye Terminator Ready Reaction Mix version 1.1 (Life Technologies, USA), 1.5 μl of ABI PRISM BigDye Terminator 5X Sequencing Buffer version 1.1, 3.1 (Life Technologies, USA), and 4 μl water. For level of sensitivity assay, 1.0 μМ V600E-SP and 0.5 μМ V600-SP were used.
7. Sequencing condition consisted of 25 cycles of 96 °C for 15 s and 60 °C for 2 min 45 s.
8. Sequencing products were separated from unincorporated dye terminators using CleanSEQ according to manufacturer’s instruction (Beckman Coulter, USA).
9. Samples were then dried in a Speed Vac (DNA 120, ThermoSavant, USA) and re-suspended in 20 μl of Hi-Di formamide. Samples were diluted 1:10 with Hi-Di formamide prior to analysis.
10. Samples were analyzed by capillary electrophoresis using ABI PRISM Genetic Analyzer 3130. Application of Tumorplex™ was tested by detecting *BRAF* V600E sequencing analysis software v6.0 (Thermo Fisher Scientific, USA).

## Method validation

**Fig. 1:**
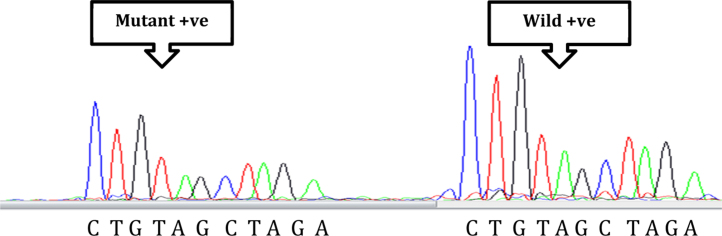
Electropherogram showing both nucleotide sequence generated by mutant sequencing primer and wild type sequencing primer.

The method includes simultaneous detection of *BRAF* V600E mutant and its wild-type. To show specificity of the method, mutant V600E and wild-type V600 templates were sequenced with V600E and V600 sequencing primers simultaneously, and separated by capillary electrophoresis. When both sequencing primers were tested with DNA template carrying 50% mutant and 50% wild-type allele in the same reaction, the result showed two identical sequences, (CTGTAGCTAG), generated by mutant V600E-SP (AATAGGTGATTTTGGTCTAGCTACAGT) and wild-type V600-SP (AATAGGTGATTTTGGTCTAGCTACAGA). However, based on the molecular weight of the sequencing primers the corresponding truncated molecules migrated differently creating a separation between the two short sequences (Fig. 1). The mutant sequence migrated first, followed by the signal generated from the heavier wild-type sequencing primer.

**Fig. 2:**
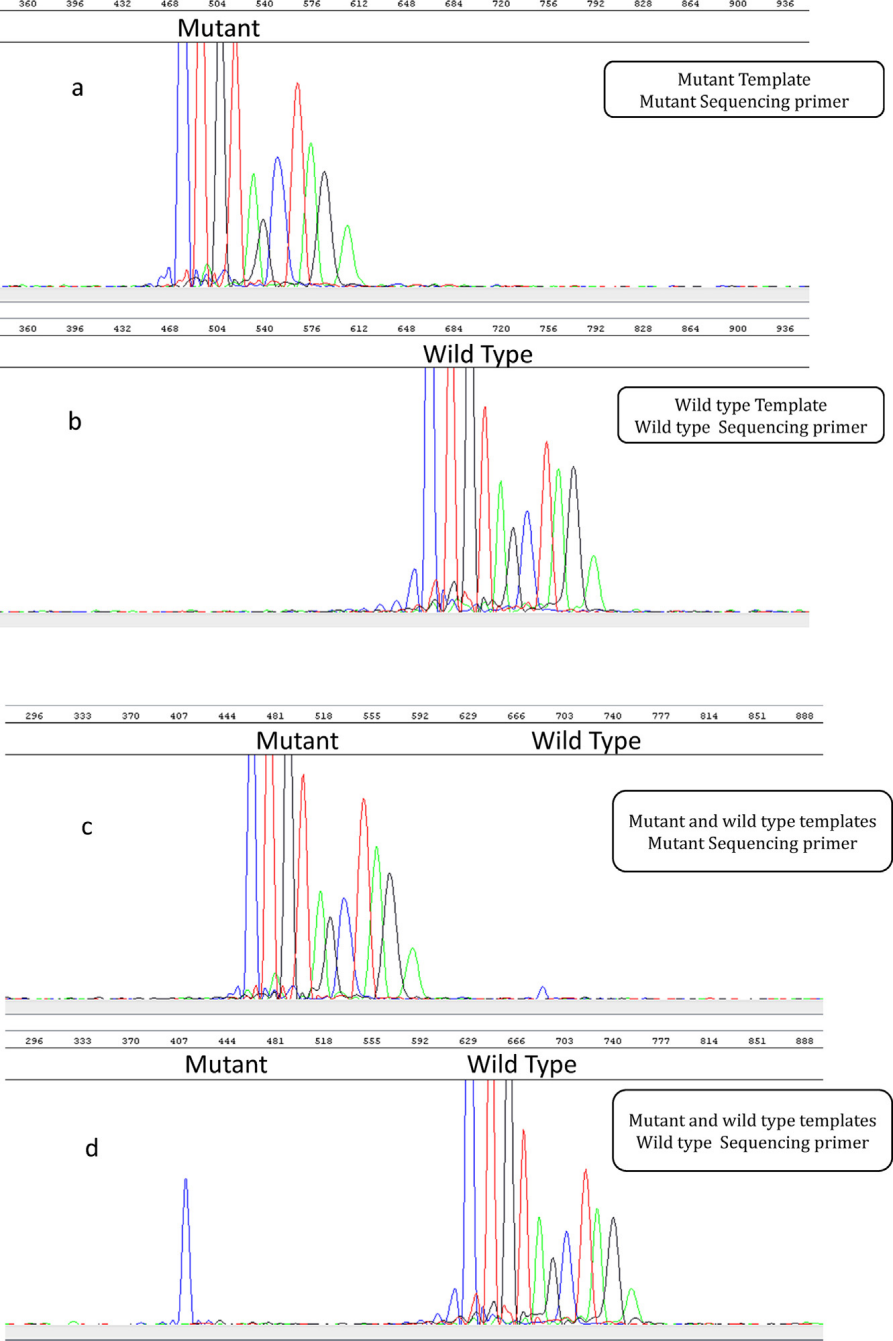
Showing primer specificity, (a) mutant SP with mutant template, (b) wild type SP with wild type template, (c) mutant and wild type SP with mutant template, (d) mutant and wild type SP with wild type template.

Primer specificity was performed by testing both the mutant and wild-type sequencing primers with mutant template and wild-type template in combinations and individually. When the mutant sequencing primer was tested with mutant template, only mutant signal was generated (Fig. 2a), but no signal was generated with wild-type template (data not shown). Similarly, when wild-type sequencing primer was tested with wild-type template identical signal was generated at the expected data point on the electropherogram (Fig. 2b), but no signal was generated when mutant template was tested used (data not shown). When both the sequencing primers were tested with mutant template, there was only mutant signal on the electropherogram and no wild-type signal (Fig. 2c). When both the sequencing primers were tested with wild-type template, there was only wild-type signal on the electropherogram and no mutant signal (Fig. 2d).

**Fig. 3:**
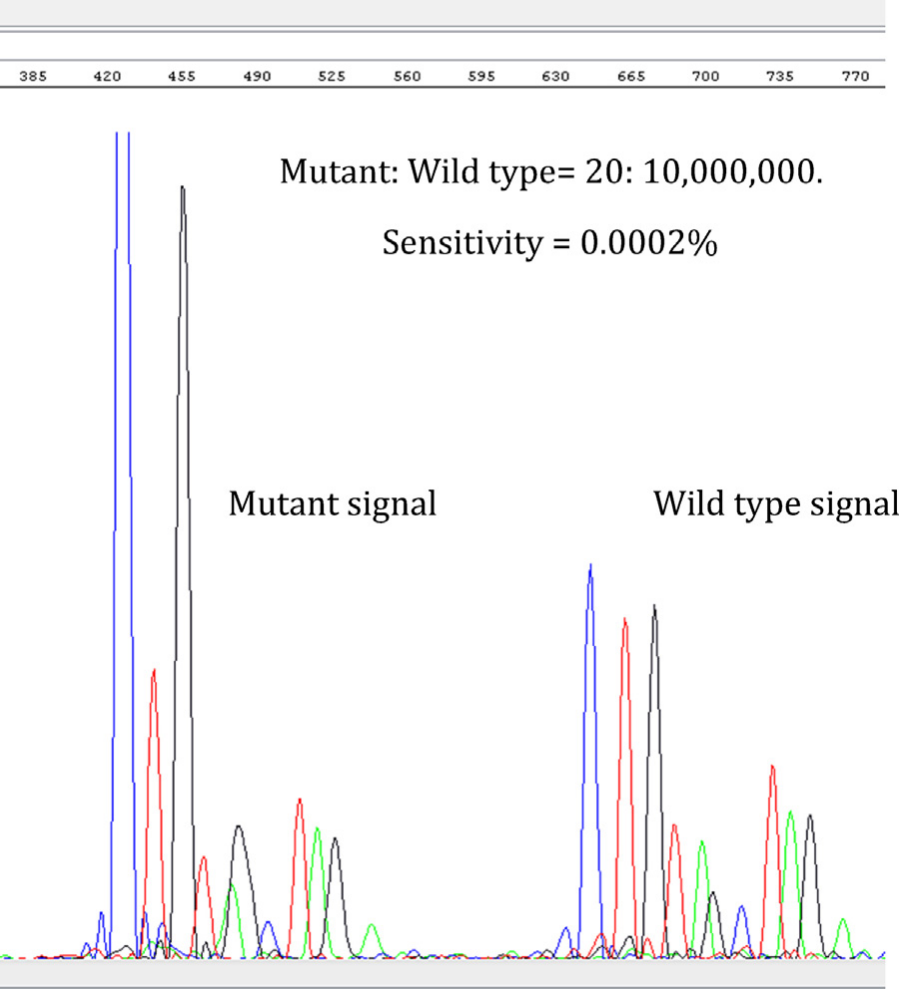
Electropherogram showing mutant and wild type signal.

To determine LLOD for V600E, serial dilutions of the V600E template were set up using human gDNA. The expected nucleotide sequence was detected with the dilution carrying 20 GE (Table 1A). To determine the level of sensitivity, experiments were carried out using different ratios of V600E to V600 templates. The results showed that mutant V600E sequence detected when the GE ratio was as low as 20:1 × 10^7^ (mutant to wild-type) (Table 1B; Fig. 3).

## Additional information

Formalin fixed paraffin embedded (FFPE) tumor biopsies are routinely analyzed for specific genetic mutations to help identify tumors, select chemotherapy, and determine prognosis of the disease [1]. However, this analysis is challenged by a number of technical problems, which affects its use for genetic analysis, such as DNA sequencing. These difficulties include: sample size in biopsies – especially in fine needle aspiration (FNA), clonal heterogeneity, a need for a built-in internal control, and the necessity to increase the cancer content by cell enrichment before downstream molecular analysis [1], [2], [3]. In order to meet these technical challenges, we have modified the Sanger sequencing platform to simultaneously detect the presence of both mutant and wild-type alleles in a single reaction without cell enrichment [4].

The molecular basis of Tumorplex™ uses a pair of PCR primers to amplify the segment of human gDNA containing the mutation site. These PCR primers generate amplicons, some carrying the mutant allele and others carrying the wild-type allele. These two sets of amplicons are then simultaneously sequenced, using two unique sequencing primers: one carrying the mutant nucleotide at its 3′ end that recognizes the mutant allele, and the other corresponding wild-type nucleotide, which recognizes the wild-type allele. The electropherogram will show two sets of identical nucleotide sequences. Based on the molecular weight differences of the respective sequencing primers, these two sets of sequences are spatially separated by capillary electrophoresis [5].

*BRAF* V600E mutation (c.1799T>A, which results in p.Val600Glu amino acid change) [6] is associated with thyroid, melanoma and colorectal cancers. Its detection is vital in diagnosis and treatment modalities [7], [8], [9], [10], [11], [12]. The *BRAF* gene encodes a kinase found in the signal transduction pathway from Ras to MEK 1/2, which plays a key role in the proliferation of melanocytes [13]. Hence, *BRAF* V600E is a marker for companion diagnosis, e.g., Melanoma patients treated with vemurafenib [14], as well as a marker for aggressive papillary thyroid cancer [15], [16]. To test the ability of Tumorplex™ to identify a mutant allele in a mixed population, we used the *BRAF* V600E SNP mutation for detection against its wild-type.

The Tumorplex™ assay offers four unique advantages over current detection methods including FFPE/Sanger sequencing methods. First, the Tumorplex™ assay generates an endpoint, with distinct nucleotide sequences, allowing for instant verifiable accuracy. Any aberrant annealing of sequencing primers would generate a sequence different from the expected sequence on the electropherogram. Second, the absence of a *BRAF* V600E template showed only the sequence generated by the wild-type sequencing primer. Hence, the wild-type acts as an amplification control, reducing false negatives. Third, since the assay detected *BRAF* V600E mutation at a very low LLOD, this decreases the chance of missing low abundant clonal cancer cells in biopsy samples. Fourth, these experiments demonstrate the ability of the Tumorplex™ technology to detect *BRAF* V600E from small amounts of extracted DNA without wild-type suppression. This capability eliminates the cost and variability of performing traditional cell enrichment, as well as eliminating the use of stains that may affect downstream amplification steps. We plan to continue to evaluate this method with clinical samples.

In summary, the Tumorplex™ platform technology has the ability to detect specific somatic mutational changes from small amounts of sample carrying heterogeneous gDNA and at substantially higher sensitivity than currently available methods.

## Figures and Tables

**Table 1:** LLOD and sensitivity analyses for V600E; positive sequence call (+) and negative sequence call (−).

(A)	
Lower limit of detection	
Copies	V600E
1560	+
195	+
20	+
10	–
2	–
Negative control	–

(B)		
Sensitivity		
V600E		
GE (V600E:V600)	V600E	V600
20:1.25 × 10^5^	+	+
20:2.5 × 10^5^	+	+
20:5 × 10^5^	+	+
20:1 × 10^6^	+	+
20:1 × 10^7^	+	+
Negative control	−	−

## References

[1] Lade-Keller J., Rømer K.M., Guldberg P., Riber-Hansen R., Hansen L.L. (2013). Evaluation of BRAF mutation testing methodologies in formalin-fixed, paraffin-embedded cutaneous melanomas. J. Mol. Diagn..

[2] Moore D.A., Saldanha G., Ehdode A., Potter L., Dyall L. (2013). Accurate detection of copy number changes in DNA extracted from formalin-fixed, paraffin-embedded melanoma tissue using duplex ratio tests. J. Mol. Diagn..

[3] Kapp J.R., Diss T., Spicer J., Gandy M., Schrijver (2014). Variation in pre-PCR processing of FFPE samples leads to discrepancies in BRAF and EGFR mutation detection: a diagnostic RING trial. J. Clin. Pathol..

[4] Vinayagamoorthy T., Luevano D., Hodkinson R. (2013). Allele Specific Multiplex Sequencing (ASMS) for the Detection of Mutations in Heterogeneous Cell Populations (Solid Tumors).

[5] Vinayagamoorthy T., Mulatz K., Hodkinson R. (2003). Nucleotide sequence based multi-target identification, MultiGEN. J. Clin. Microbiol..

[6] Davies H., Bignell G.R., Cox C., Stephens P., Edkins S. (2002). Mutations of the BRAF gene in human cancer. Nature.

[7] Benlloch S., Payá A., Alenda C., Bessa X., Andreu M. (2006). Detection of BRAF V600E mutation in colorectal cancer comparison of automatic sequencing and real-time chemistry methodology. J. Mol. Diagn..

[8] Weyant G.W., Wisotzkey J.D., Benko F.A., Donaldson K.J. (2014). BRAF mutation testing in solid tumors. A methodological comparison. J. Mol. Diagn..

[9] Qu K., Pan Q., Zhang X., Rodriguez L., Zhang K. (2013). Detection of BRAF V600 mutations in metastatic melanoma comparison of the Cobas 4800 and Sanger sequencing assays. J. Mol. Diagn..

[10] Kristensen T., Clemmensen O., Hoejberg L. (2013). Low incidence of minor BRAF V600 mutation-positive subclones in primary and metastatic melanoma determined by sensitive and quantitative real-time PCR. J. Mol. Diagn..

[11] Board R.E., Ellison G., Orr M.C.M., Kemsley K.R., McWalter G. (2009). Detection of BRAF mutations in the tumour and serum of patients enrolled in the AZD6244 (ARRY-142886) advanced melanoma phase II study. Br. J. Cancer.

[12] Greaves W.O., Verma S., Patel K.P., Davies M.A., Barkoh B.A. (2013). Frequency and spectrum of BRAF mutations in a retrospective, single-institution study of 1112 cases of melanoma. J. Mol. Diagn..

[13] Buscà R., Abbe P., Mantoux F., Aberdam E., Pessonnaux C. (2000). Ras mediates the cAMP-dependent activation of extracellular signal-regulated kinases (ERKs) in melanocytes. EMBO J..

[14] Chapman P.B., Hauschild A., Robert C., Haanen J.B., Ascierto P. (2011). Improved survival with vemurafenib in melanoma with BRAF V600E mutation. N. Engl. J. Med..

[15] Ciampi R., Nikiforov Y. (2007). RET/PTC rearrangements and BRAF mutations in thyroid tumorigenesis. Endocrinology.

[16] Cohen Y., Rosenbaum E., Clark D.P., Zeiger M.A., Umbricht C.B. (2004). Mutational analysis of BRAF in fine needle aspiration biopsies of the thyroid: a potential application for the preoperative assessment of thyroid nodules. Clin. Cancer Res..

