# Corrigendum to “Detection of *BRAF* mutations from solid tumors using Tumorplex™ technology” [MethodsX 2 (2015), 316–322]

**DOI:** 10.1016/j.mex.2016.01.006

**Published:** 2016-02-02

**Authors:** Jacob Yo, Katie S.L. Hay, Dilanthi Vinayagamoorthy, Danielle Maryanski, Mark Carter, Joseph Wiegel, Thuraiayah Vinayagamoorthy

**Affiliations:** MultiGEN Diagnostics LLC, 854 Paragon Way, Rock Hill, SC 29730, United States

The authors regret that the original version of the Method details contained an error. The correct paragraph is re-produced below

## Method details

### Primer design

A set of PCR primers BRAF-UP: 5′-GAA GAC CTC ACA GTA AAA ATA GGT GAT TTT GGU C-3′ and BRAF-LP: 5′-TCAATGACTTTCTAGTAACTCAGCAGCATCTC-3′ were used to amplify a region of human gDNA encompassing the BRAF V600 locus. The amplified product was 180 base pairs (bp). Two sequencing primers were designed to recognize the respective single nucleotide at their 3′ end independently, one for BRAF mutant V600E and the other for BRAF wild type V600. These two sequencing primers differed in two respects: the nucleotide at their 3′ end and the respective molecular weight. Allele specific nucleotide at their 3′ end determined their respective specificity of the two sequence primers. e.g., BRAF V600E mutant harbors a deoxythymidine and V600 wild-type carries a deoxyadenosine. The different molecular weights of the allele-specific sequencing primer separated the truncated molecules generated from the mutant sequencing primer from the wild type sequencing primer. Allele-specific sequencing primers used were (mutant) V600E-SP: 5′-AAC TGA TGG GAC CCA CTC CAT CGA GAT TTCT-3′ and (wild type) V600-SP: 5′-weighted-AAC TGA TGG GAC CCA CTC CAT CGA GAT TTC A-3′.

The authors would like to apologize for any inconvenience caused.

